# “Impact of leadership styles on innovative performance of female leaders in Pakistani Universities”

**DOI:** 10.1371/journal.pone.0266956

**Published:** 2022-05-12

**Authors:** Uzma Sarwar, Samina Zamir, Kiran Fazal, Yang Hong, Qi Zhan Yong

**Affiliations:** School of Education, Shaanxi Normal University, Xi’an, 710062, PR China; American University of Sharjah, UNITED ARAB EMIRATES

## Abstract

Leadership plays a significant role in the performance of individuals and organizations. This paper investigates the impact of leadership styles on the innovative performance of female leaders in Pakistani Universities using a survey approach. This paper aimed to (a) discover a leadership style practiced by females and (b) discover the relationship between leadership style and the innovative performance of female leaders. Several female leaders, including faculty members, heads of departments, deans, coordinators, and directors, from public and private universities of Punjab (a province of Pakistan), were involved in this study. A sample of one hundred female leaders was selected using a multistage sampling method. In the first stage, five public and five private sector universities were selected through a simple random method. In the second stage, ten female leaders (five from each of the social science and basic science departments) were selected from each university through a purposive sampling method. The researchers adopted a reliable instrument to collect the survey data. The collected data were analyzed using SPSS (Version 26). Mean scores and Pearson correlation coefficients were used to discover the relationship between various variables. The investigation revealed that most female leaders practice the transactional leadership style in their universities. This study also discovered a moderate positive relationship between both leadership styles, namely transactional and transformational, and innovative performance. The study recommends that various workshops and seminars may be conducted to increase the practices of both leadership styles to enhance innovation in Pakistani Universities.

## 1. Introduction

Organizations are operating in a dynamic environment in this era of technological developments, with rapid technological progress demanding organizations contribute creativity and innovation to their products and services. The importance of employee creativity for innovation has been widely stressed in literature [1]. It has been proven that encouraging individual innovation is critical for businesses to be economical and survive in the marketplace. Additionally, numerous firms are also continuously observing and adopting new techniques to motivate their personnel to be more creative and come up with new ideas [2]. One approach is through leadership, regarded as one of the most important variables influencing employee creativity and organizational innovation. Leadership has also been identified in terms of a significant component of corporate innovation [3].

Leadership has played a significant role in human history, and leadership style played a vital role in building a conducive working environment and culture inside organizations. The leadership style, in particular, motivates people to struggle eagerly to achieve the organization’s goals [4]. A leader’s role is to impart knowledge by displaying learning behavior to motivate staff to develop fresh ideas. Hurduzue [5] argued that an effective leadership style might develop various competencies of individuals working in an organization. Leaders and their practiced leadership styles have been recognized as the most explored subjects in the literature. A leadership style is documented as the leader’s behavior and strategies, formulation and implementation of strategies for providing a vision and a runway to achieve that vision through utilizing available resources. Every leader working in any organization has a style of leadership. Individuals use their leadership styles considering the nature of the situation, and there are many differences in the leadership styles practiced by individuals.

Moreover, there are many leadership theories, and each approach demarcates the components of leadership according to their concepts [6]. Traditional leadership theories include the Great Man Theory (1840–1910), trait theory (1910–1948), behavioral theory (1950–1970), and contingency theory (1967–1990), while modern leadership philosophies include transformational and transactional leadership (1985–2010) [6]. The transactional and transformational leadership styles have stimulated the curiosity of countless scholars and researchers [7,8].

In this competitive atmosphere, an organization’s primary purpose is to improve employee work performance to improve productivity and the quality of life [9]. Effective leadership and better collaboration between leaders and workers are the key drivers of organizational success. The primary responsibility of a leader is to support their organization by exhibiting positive behavior and adopting an appropriate strategy to achieve the organization’s mission in a short period. There is much evidence regarding the importance of leaders and their leadership style and its association with job performance, commitment, and organization’s performance. Many studies have discovered that leadership styles are essential determinants of job performance. Several investigations covering leadership styles have been carried out in developed as well as developing countries [10–14].

Innovation is the process that enables a person to generate and apply new ideas or innovation to perform better in their job and meet organizational goals, which is known as innovative job performance [15]. According to [16], innovation is the ability of an individual to perceive issues and provide unique ideas for a solution. It is the competency to implement their ideas appropriately [16]. Many factors influence innovation and the innovative performance of employees. These factors include an individual’s motivation, personality, and organizational support [17,18]. According to [19], innovation is a multi-stage process. Leaders play their role in implementing this multi-stage process in businesses and turn ideas into better products and services to compete, progress, and sustain in the market. An organization’s products, services, manufacturing and distribution systems, organizational procedures, marketing, and design processes are different application areas where innovation can be found.

Employees’ innovative behaviors and activities are becoming increasingly important in enhancing the excellence and performance of systems and institutions, demanding the measurement of these behaviors and actions. Most of the educational systems recognized the importance of assessing innovation and innovative performance in education. They have given a top priority to this subject with the collaboration of critical international agencies actively involved in promoting innovation in this field [20–22]. The OECD [23] recently proposed a survey for measuring innovation in the education sector to its members’ countries. The European Commission has expressed strong support for this initiative. The initiative proposed by OECD [23] demands innovation and innovative performance in education at a global level.

Despite the importance of an individual’s leadership style and its impact on job performance in terms of innovation, there is limited research in the literature regarding discovering the effect of transactional and transformational leadership styles on the innovative performance of female leaders in Pakistani Universities. Keeping given the enlightenment above about the importance of leadership style and its association with innovation performance, this study aims to discover the impact of leadership style on the innovative performance of female leaders’ working in the universities of Pakistan.

## 2. Literature review

Burns [24] was the first to propose the idea of transformational leadership, and later [25] extended it by explaining how transformational leadership contributes to inspiring employees to work diligently and achieve the organization’s goals. Initially, three characteristics of transformational leadership, namely idealized influence, intellectual stimulation, and individualized consideration, were used to measure the leader’s qualities. Later, another element of transformational leadership, inspirational motivation, was introduced by [26]. Subsequently, [26] further explored four characteristics of transformative leadership, including idealized influence, inspirational motivation, intellectual stimulation, and individual consideration. The idealized influence deals with how leaders can inspire their workers to follow them as their role models. Inspirational motivation deals with the leader’s strategies for stimulating and motivating their employees outside of their presumptions to attain organizational and personal goals [26]. Intellectual stimulation refers to the leader’s approaches to encourage employees to renew their minds and look outside the box during problem-solving activities. The individual consideration refers to the leader’s personal qualities and gives close attention to each of their employees, listening to their problems and providing help. Several studies have investigated the association of transformational leadership towards workers’ creativity, commitment, and innovative performance [27] and discovered a positive association between leadership styles and employees’ creativity, dedication, and performance. These findings also contributed to a better understanding of workforce management for creativity and innovation. Beyond self-interest, transformational leaders focus on employees, companies, and societies [28]. Employees are motivated to work for long hours and produce more than estimated by the leaders who practice transformational leadership style [26]. According to an investigation, transformational leaders can stimulate and urge their followers to go above and beyond their typical expectations and develop a strong sense of commitment and togetherness among employees to enhance performance [29]. Transformational leadership, according to past research, had a favorable influence on the success of the employees and well as future promotion [30,31].

According to Burns [24], as referenced in [32], transformational leadership encourages, inspires, and motivates followers to innovate and create change that will help grow and shape the firm’s future success. According to [32], leaders must display transformative leadership behaviors to gain their people’s trust, loyalty, and respect. Workers are motivated to go above and beyond what is anticipated due to the transformational leadership style. Through transformative leadership behaviors, employees can understand the importance of job outcomes. Based on the significance of the job’s results, employees work towards sacrificing self-interest for the company’s sake and activating their higher-order necessities. Leaders practicing transformative leadership styles, according to [33], contribute to enhancing workers’ performance, whereas leaders practicing transactional leadership styles produce predictable outcomes.

In comparison to other styles of leadership, the transformational leadership style has gained more comprehensive favor among leadership researchers due to its unique ways of motivating employee innovation [34–38]. Furthermore, researchers believe that the transformational leadership style significantly impacts workers’ creativity and is well-suited to identifying new opportunities and developing organizational competencies. Transformational leaders boost employees’ confidence and values, and because of this, the worker’s output exceeds expectations [39]. Transformational leaders exchange knowledge and inspire their staff to develop new ideas to boost an individual’s creativity. Leaders practicing transformational style in their administration course inspire people to initiate unique thinking to generate innovative solutions through intellectual inspiration. According to [40], one of the primary components of inspirational motivation is formulating and articulating a shared vision that encourages people towards creativity. The traits of these transformative leaders can assist employees in performing creatively. Employees who are innovative and creative are a valuable source of new ideas. Transformational leaders create an environment where individuals are encouraged to learn, share, and try out new ideas. Thus, transformational leaders of various organizations focused on the encouragement of an employee towards creativity to turn ideas into new products and services for getting a competitive advantage in the marketplace [41].

Wilkes et al. [42] and Jiang et al. [31] reported that transformational leadership had gained traction as a powerful leadership style that all leaders should employ. As a result of this leadership behavior, employees are anticipated to be motivated and inspired to go beyond the intended objectives and to modify their behaviors and beliefs [43]. Previous research discovered a strong association between transformative leadership and workers’ performance [31,44,45]. Likewise, Wang et al. [45] discovered that transformative leadership is an essential predictor of workers’ contextual and job performance. Additionally, [44] explored the role of transformational leadership style on employees’ innovative performance and discovered a significant relationship between the two variables. They reported that all four characteristics of transformational leadership, including inspiring motivation, intellectual stimulation, individualized consideration, and idealized influence, were significant determinants of employees’ contextual performance and success.

The transactional leadership style is recognized and used in a typical managerial function. During the end of the 1970s, leadership theory research focused on improving organizational performance, and most executives adopted a style of leadership known as a transactional leadership style. The transactional leadership style is still the most well-known leadership technique in today’s enterprises. Transactional leaders lead by providing particular incentives and motivating others by trading one thing for another [46]. Employee responsibilities and job requirements are clarified by transactional leaders, who begin structure, give rewards, and exhibit employee attention. Transactional leaders enjoy ensuring that things operate quickly and efficiently, and they generally adhere to established rules and regulations. As a result of their position, the transactional leader implements policies, maintains the status quo, and gains authority. According to [47], transactional leadership emphasizes the paths necessary for managing the status quo and maintaining the day-to-day procedures of a business. This leadership style does not emphasize identifying the organization’s directional goals and how workers can work toward achieving those goals, increasing their productivity in alignment with these goals, thus increasing organizational profitability. Transactional leadership prioritizes control over adaptation. Transactional leadership is limited because it does not consider the jam-packed issue, workers, or the organization’s future when awarding rewards. Transactional leadership is almost ineffective in today’s competitive climate when innovation and originality are vital requirements for business success [48,49].

Employee morale standards are thought to be compatible with transactional leadership. It raises the employee’s awareness of ethical issues while generating energy and resources. Employees are driven by transactional leadership because it appeals towards workers’ self-interest and provides incentives and benefits. Transactional leadership is a type of leadership that focuses on the interactions between a leader and their workforce. Employees who comply with the leader’s requests are rewarded [33,32]. As a result, transactional leaders’ styles and behaviors are centered on the contractual agreements of the employees and the benefits that come with them [24]. According to Northouse [33], transactional leadership is based on the compensation and fulfillment of a contractual responsibility by employees. Transactional behaviors include contingent reward, passive management by exception, and passive management by exception, according to [25], as quoted by [32]. Additionally, [26], as mentioned by [32] added another transactional behavior known as active management via an exception. Moreover, transactional leadership, on the other hand, does not promote employee engagement [32].

According to [25], the theory regarding transactional leadership comprises three dimensions: (a) contingent reward, which focuses on clarifying the labor necessary to get the reward, and incentive and contingent reward to influence motivation. These leaders aid people in exchange for their efforts. They set clear expectations and offer praise when objectives are met, (b) management by exception (passive), which deals with the utilization through contingent punishments and other corrective actions in response to worker’s deviations from satisfactory performance criteria, and (c) management by exception (active), which deals through monitoring employees and taking action when they are not performing as per defined criteria. In addition, it means that a leader takes notice of & workers’ deviation from the rules and regulations and takes corrective actions [50]. Leaders who practice the passive management-by-exception strategy wait for the problem to occur before intervening [8]. When a leader pays or punishes their employees based on how well they perform, this is known as the transactional leadership style [51]. As a result, a transactional leader’s priorities are to encourage individual’s towards their self-interest through announcing incentives [52]. According to transactional leadership theory, compensation is recognized as an essential element for motivating employees. As a result, two types of transactional leader tactics for managing individuals have emerged: the first one is contingency reward, and the second one is management-by-exception [26].

According to [34], leaders practicing transactional style focus to inspire and empower their followers to take chances and own their outcomes, which connects the creative environment and encourages followers towards creative work. In addition, transformational leaders encourage their people to produce unique and innovative ideas by encouraging them to find alternate ways to complete their tasks. According to [34], leaders practicing transactional style have to value creativity and innovative work and support innovation to influence followers’ creativity and innovation favorably. In addition, transformational leaders encourage people to develop new ideas and apply creative problem-solving strategies to boost employee creativity and achieve organizational innovation. To motivate and promote their innovative performance, transformational leaders set performance standards and display trust in their workers.

The transactional leadership style primarily focuses on supervision, organization, and performance of employees. This form of leadership was appeared through the industrial revolution and considered a source of economic advantage. Transactional leadership has some typical management strategies such as efficiency goals, economies of scale, and quality differentiation. Transactional leaders always focus on performance-relevant tasks and goals [53]. Moreover, [24] highlighted the role of transactional leadership, such as leaders practicing this style always stimulate compliance by followers through reward and punishment system. Transactional leaders practice reward and punishment systems for keeping workers motivated for the short term. Leaders practicing the transactional method dislike transformational leaders, as they are not trying to influence the future. Therefore, it can be said that the central theme of transactional leadership is to provide valuable exchange rewards for the achievement of the company’s goals.

According to [54], the transactional leadership style is mainly considered adequate during situations of crisis and emergencies. This style has two recognized mechanisms; the first one is a contingent reward, and the second is management by exception. The first element is an efficient and constructive relationship between leaders and subordinates. The subordinates of the company meet specific goals of the company and achieve bonuses, merits, or recognition within the company. These rewards are purely based on an agreement among the leaders and subordinates. While the second component, transactional leadership, is management by exception. According to this component, a leader can be active or passive as a dynamic leader always observing to measure the performance of an employee, whereas, a passive leader only assesses after the completion of work and will inform you of issues after they have happened [55].

Previous researchers have discovered a positive and negative relationship between transactional leadership behaviors and employee performance [56–58]. According to a study conducted by [56] among banking employees, transactional leadership behaviors positively and significantly impact employee performance and productivity. The working environment affects employee performance. According to [58], transactional leadership has a solid and positive relationship with employee performance. Contingent rewards have a positive and immediate impact on employee performance, according to [59], through giving a safe and enjoyable working environment, autonomy, and leadership support. Yang and Yang [60] explained that rivalry and competitiveness could affect leadership effectiveness. When there is no competition, transactional leadership will significantly impact workers’ innovation and performance.

On the other hand, [61] discovered that transactional leadership was not a strong predictor of employee performance. Educators and scholars have also stated that no single leadership style is beneficial in all situations [62]. An individual’s leadership style should be appropriate for the context or circumstance in which they interact with his or her staff, described by [63].

Furthermore, some investigations have observed the impact of different leadership styles on employee performance, creativity, and innovation [64]. The majority of them have contributed to the literature and provided important insights. Moreover, the impact of transformational leadership in enhancing creativity was the main direction of these investigations. Because of its significant effects on employees, transformational leadership has become a well-known type of leadership among researchers. The favorable and noteworthy impact of transactional leadership in predicting creativity has also been shown in recent studies [35,36,65]. Furthermore, according to the investigation’s outcome of [66], transformational leadership has a more significant impact on employee performance as compared to transactional leadership.

In their study, [67] found a good and substantial association between transformational leadership style and inventive employee performance. Furthermore, according to a recent South African study, inspiring motivation and intellectual stimulation are positively related to innovation and innovative performance [52]. Moreover, [68] found that intellectual stimulation had a favorable impact on innovation. In addition, transformational leaders develop strong relationships with their staff, which leads to improved job performance [69].

## 3. Statement of the problem

Leadership style plays an essential role in the progress of all organizations. There is a lack of understanding about the leadership style practiced by the leaders working in the educational sector. In Pakistani’ culture where males dominate most of the fields, including education, compared to females. Literature has much evidence about leadership and its impact on leaders’ performance, but there is a shortage of studies in the available literature that investigated the impact of transactional and transformational leadership styles on innovative performance, especially female leaders’ focusing Pakistani educational field.

## 4. Research objectives

The research objectives were to:

1. Explore which and to what extent a leadership style is practiced by female leaders in Pakistani Universities?
2. Investigate the relationship between transactional leadership style and innovative performance of female leaders in Pakistani Universities?
3. Discover the relationship between transformational leadership style and innovative performance of female leaders in Pakistani Universities?

## 5. Research methodology

The present investigation aims to discover the relationship between transactional and transformational leadership styles and the innovative performance of female leaders working in public and private universities of Pakistan. Based on the abovementioned purpose, the current study utilized a correlational investigation. Females working as deans, heads of departments, directors, and senior faculty members of public and private universities located in the Punjab province of Pakistan constituted the population of this investigation. A sample of one hundred female leaders was selected using the multistage sampling method. Ten universities (5 public and five private sectors) were chosen in the first stage through the simple random process. At the second stage, ten female leaders (5 from the social science department and five from the basic science department) from each university were chosen through the purposive sampling method. An instrument in the shape of a questionnaire based on a five-point Likert scale was adopted to collect quantitative data from the female leaders. The questionnaire has four sections: section one was about demographic information, section two was about the items of transactional leadership style, section three was about the items of transformational leadership style, and section four was about the items of innovative performance. In this way, there were 33 items in the questionnaire. Sections two and three of the questionnaire were adopted [26], and the first author herself developed the remaining sections. The face, content, and construct validity of the research instrument were established by obtaining expert opinions from the supervisor and experts. Later on, pilot testing was carried out to confirm the reliability of the questionnaire.

Data collected in the pilot testing phase was assessed in SPSS, and a scale reliability test was used. The results of the scale reliability test (α = .896) proved that the instrument is reliable and suitable for data collection. Later on, the actual data were collected with a research assistant’s help and an online technique using Google Form. The collected data were analyzed in SPSS (Version 26). Descriptive and inferential statistics were used to explore the level of agreement and relationship between independent and dependent variables. The outcomes of the data analysis and research model used for this investigation is presented below.

### 5.1 Research framework

**Fig 1:**
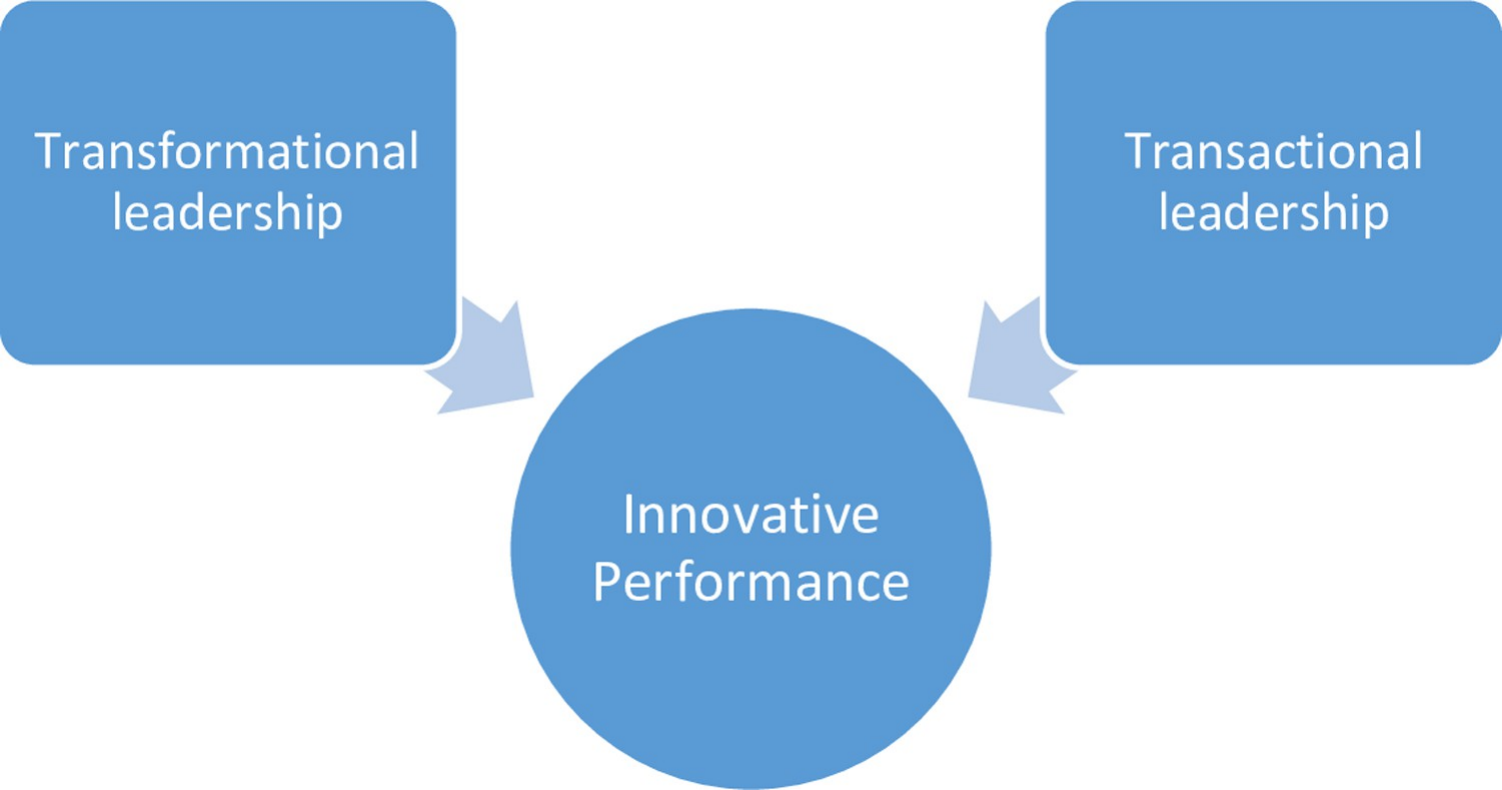
Research framework.

Based on the previous discussion, a conceptual framework was developed as shown in Fig 1.

### 5.2 Ethical considerations

This research study was carried out in the Punjab Province of Pakistan. The researcher met ethical requirements at each step of the research process. A permission letter was obtained from the supervisor and the Ethics Committee members of the school of education at Shaanxi Normal University, Xi’an, China. This research involves the completion of a questionnaire that comprises questions related to leadership styles and innovative performance. The participants of this study include deans, heads of departments, directors, coordinators, and senior faculty members of public and private universities. The researcher enclosed the participant consent form with the questionnaire. All the study participants were informed about the purpose of the research, and the necessary permission was taken before data collection. The information collected was used only for academic purposes and kept confidential.

## 6. Results/Findings

The data were collected from female leaders working in Pakistani Universities through a questionnaire. Descriptive and inferential statistics were used to discover the relationship between independent and dependent variables. Descriptive statistics were used to measure the female leaders’ level of agreement towards the practice of transformational and transitional leadership styles. The below criteria were adopted to measure the level of agreement or disagreement.

Table 1: shows the criteria adopted for assessing the level of practicing females’ leadership styles.

**Table 1:** Criteria to assess females’ level of leadership style and innovative performance.

Mean Scores (*M*)	Perceiving degree
Less than 1.8	Very low
1.8 to 2.5	Low
2.6 to 3.5	Moderate
3.6 to 4.2	High
4.3 and above	Very High

### RQ1: Which and to what extent a leadership style is practiced by female leaders in Pakistani Universities?

**Table 2:** shows that indicator wise female leaders’ perception. The statistical information demonstrated in Table 2 shows the perceptions of female leaders about transformational and transactional leadership styles and their innovative performance. The results indicate that majority of the female leaders practice transformational leadership style with a mean score of 3.79 and transactional leadership style with a mean score of 3.20. Moreover, female leaders have a high perception of innovative performance with a mean score of 4.02. Based on the results, it has been concluded that the majority of the female leaders working in Pakistani universities practice a transformational leadership style. It is worth mentioning that female leaders working in Pakistani universities exhibit a different leadership style while performing their daily activities. It means that female leaders practice transformational leadership styles which focus on employees’ idealized influence, inspirational motivation, intellectual stimulation, and individual consideration. Moreover, it is noteworthy that males are generally performing as leaders in the educational field in Pakistani culture. There is very low participation of females in administrative roles compared to males.

**Fig 2:**
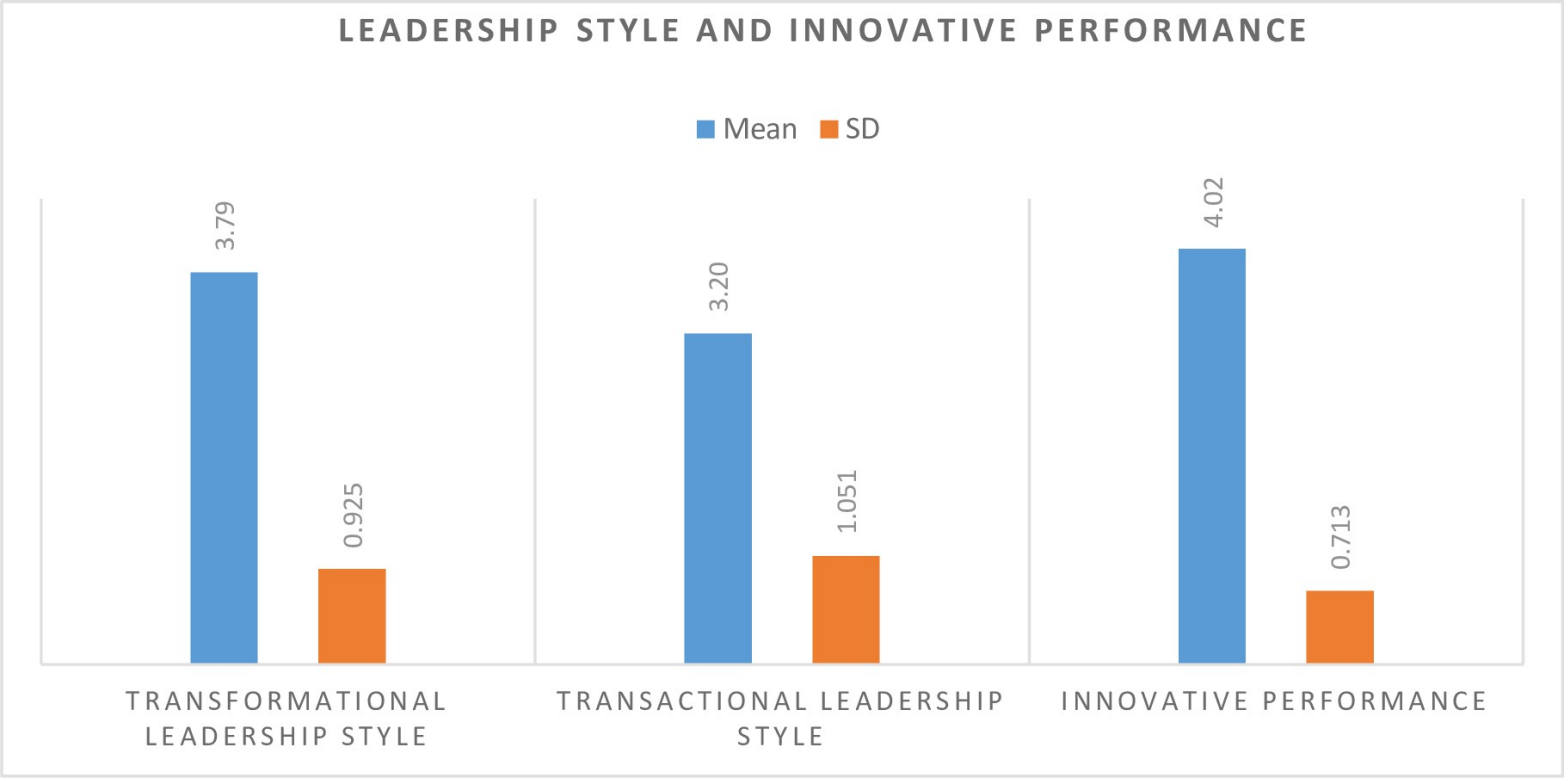
Bar chart shows that female leader practises transformational leadership style at upper lever while transactional leadership style at moderate level.

**Fig 2:** Overall Mean Scores and SDs of Female Leaders towards their Leadership Styles and Innovative Performance in Pakistani Universities.

**Table 2:** Mean scores and SDs of female leaders towards their leadership styles and innovative performance in Pakistani Universities (N = 100).

Variables	Mean	SD	Perceiving degree	Rank
Transformational Leadership style	3.79	0.925	High	2
Transactional Leadership style	3.20	1.051	Moderate	3
Innovative performance	4.02	0.713	High	1

**Table 3:** demonstrates the factor-wise means and standard deviations of both leadership styles practiced by female leaders in Pakistani Universities. According to the results, the mean score of idealized influence, inspirational motivation, intellectual stimulation, individual consideration, contingent reward, management by exception, and innovative performance are 3.74, 3.88, 3.74, 3.80, 3.43, and 3.06, respectively. All the mean values of the factors of both leadership styles were above 3.0, which confirmed that the majority of the female leader practices both leadership styles in the organizations. According to the above results, there is a significant difference according to the factor-wise analysis of both leadership styles. While in the case of innovative performance, females showed a high level of agreement. It was revealed that Pakistani female leaders understand innovation and innovative performance inside their universities. It means that the majority of the female leaders are practicing transformational leadership style compared to transactional leadership style in their routine work with a bit of modification to achieve innovative performance at their workplace.

**Table 3:** Factor wise mean scores and SDs of transactional & transformational leadership styles of female leaders (N = 100).

Factors	Mean	SD	Perceiving degree	Rank
**Transformational Leadership style**				
Idealized Influence	3.74	0.813	High	4
Inspirational Motivation	3.88	0.628	High	2
Intellectual Stimulation	3.74	0.666	High	5
Individual Consideration	3.80	0.737	High	3
**Transactional Leadership style**				
Contingent Reward	3.43	0.777	Moderate	6
Management by Exception (Active/Passive)	3.06	0.676	Moderate	7
**Innovative performance**	4.05	0.713	High	1

**Fig 3:**
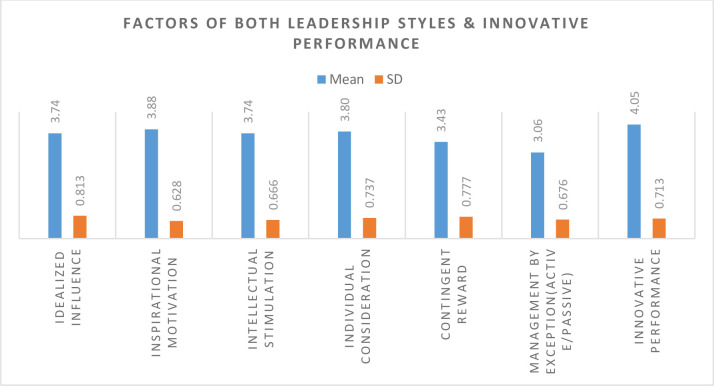
Bar chart shows the factors wise means and standards deviations of the both leadership style practised by female leaders in Pakistani universities.

**Fig 3:** Factors wise means and standards deviations of the both leadership style practised by female leaders in Pakistani universities.

### RQ: 2: Is there any significant relationship between transactional leadership style and innovative performance of female leaders?

**Table 4:** To investigate the relationship between transactional leadership style and innovative performance of female leaders working in Pakistani Universities, a test known as Pearson correlation was applied. One hundred female leaders working as deans, heads of departments, directors, faculty members, and coordinators were surveyed to discover the relationship between the independent and dependent variables. The results presented in Table 2 indicate a moderate positive relationship between transactional leadership style and innovative performance (r = .380). Moreover, the results of the p-value confirmed a statistically significant positive relationship between the independent variable (transactional leadership) and the dependent variable (innovative performance). Therefore, it is concluded that when female leaders increase the practice of transactional leadership style, their innovative performance also increases.

**Table 4:** Relationship between transactional leadership and innovative performance.

Variables		Transactional	Innovative_Performance
Transactional	Pearson Correlation		.380**
	Sig. (2-tailed)		.000
	N	100	100
Innovative Performance	Pearson Correlation	.380**	
	Sig. (2-tailed)	.000	
	N	100	100

### RQ: 3: Is there any significant relationship between Transformational leadership style and innovative performance of female leaders?

**Table 5:** Pearson r correlation test was conducted to explore the relationship between transformational leadership style and innovative performance of female leaders working in Pakistani Universities. In this regard, one hundred female leaders working as deans, heads of departments, directors, faculty members, and coordinators were surveyed to investigate the relationship between independent and dependent variables. The results presented in Table 5 show a moderate positive relationship between transformational leadership style and innovative performance (r = .472). Moreover, the results of the p-value confirmed a significant positive relationship between the independent variable (transformational leadership) and the dependent variable (innovative performance). Therefore, it is concluded that when female leaders increase the practice of transformational leadership style, their innovative performance also increases.

**Table 5:** Relationship between transformational leadership and innovative performance.

Variables		Transformational	Innovative_Performance
**Transformational**	Pearson Correlation		.472**
	Sig. (2-tailed)		.000
	N	100	100
**Innovative Performance**	Pearson Correlation	.472**	
	Sig. (2-tailed)	.000	
	N	100	100

## 7. Results and discussion

According to the findings of this study, most female leaders in Pakistani universities were practicing transformational leadership style while the minority were practicing transactional leadership style. It seems that female leaders modify their style according to the nature of the situation. As in the Pakistani culture where males dominate, females have to alter their leadership style considering their circumstances to achieve the goals of their institutions. Furthermore, female executives exhibited a positive attitude towards innovation and innovative performance. Moreover, this research discovered a moderate positive association between the transformational leadership style and innovative performance.

Similarly, this study revealed a moderate positive association between the transactional leadership style and innovative performance. Based on the results, it is perceived that female leaders working in Pakistani universities do not significantly differ between their leadership styles. Moreover, they have a high perception of innovation and innovative performance inside their organizations. These findings are consistent with those of [67], who discovered a strong positive association between transformational leadership style and innovative employee performance in their study. Another discovery by [52] showed transformational factors, such as inspiring motivation and intellectual stimulation, were positively associated with innovation and innovative performance supports the findings of this study.

Furthermore, Yasin et al. [68] found that intellectual stimulation has a beneficial impact on innovation, consistent with this study’s findings.

Similarly, several studies have demonstrated the favorable and significant influence of transactional leadership in predicting creativity [35,36], corroborating the findings of this study. Likewise [70] discovered in Pakistan that there is a significant positive association between employee performance and transformative/ transactional leaders. The study also revealed that in the case of transformational leadership, the strength of the association between leadership and employee performance was high, supporting our study’s findings. These findings are consistent with another research [71], which identifies women as more transformational rather than transactional.

## 8. Conclusion

This study concludes that most female leaders of Pakistani universities practice transformational leadership style compared to transactional leadership style. Moreover, most female leaders exhibited the factors of both leadership styles, such as idealized influence, motivation, intellectual stimulation, individual consideration, contingent reward, and management by exception (active/passive). Similarly, female leaders were inclined towards innovative performance. So, it has been revealed that female leaders have more attitudes towards practicing transformational leadership style than transactional leadership style. Furthermore, a moderate positive relationship was observed between the transformational leadership style and innovative performance.

Similarly, this investigation has revealed a moderate positive relationship between the transactional leadership style and innovative performance. It means that both leadership styles have a moderate relationship with the innovation performance of the female leaders working in the Universities of Pakistan. Based on the outcomes, this investigation recommends that various courses, workshops, and seminars may be planned to equip the female leaders in both leadership styles working in public and private sector universities to enhance innovation and innovative performance.

## 9. Limitation and study forward

This research study is limited to investigating the two leadership styles, transactional and transformational, practiced by the female leaders of Pakistani public and private sector universities located in Punjab only. A comprehensive research study can be conducted by exploring other leadership styles and provinces of Pakistan.

## Supporting information

S1 File (DOCX)

S1 Data (XLSX)
