# Supplementary material for: “Impact of leadership styles on innovative performance of female leaders in Pakistani Universities”
Source: PLoS One. 2022 May 12;17(5):e0266956. doi: 10.1371/journal.pone.0266956 (PMC9098018; doi:10.1371/journal.pone.0266956)
Supplement: S1 File — (DOCX) [file pone.0266956.s001.docx]

**
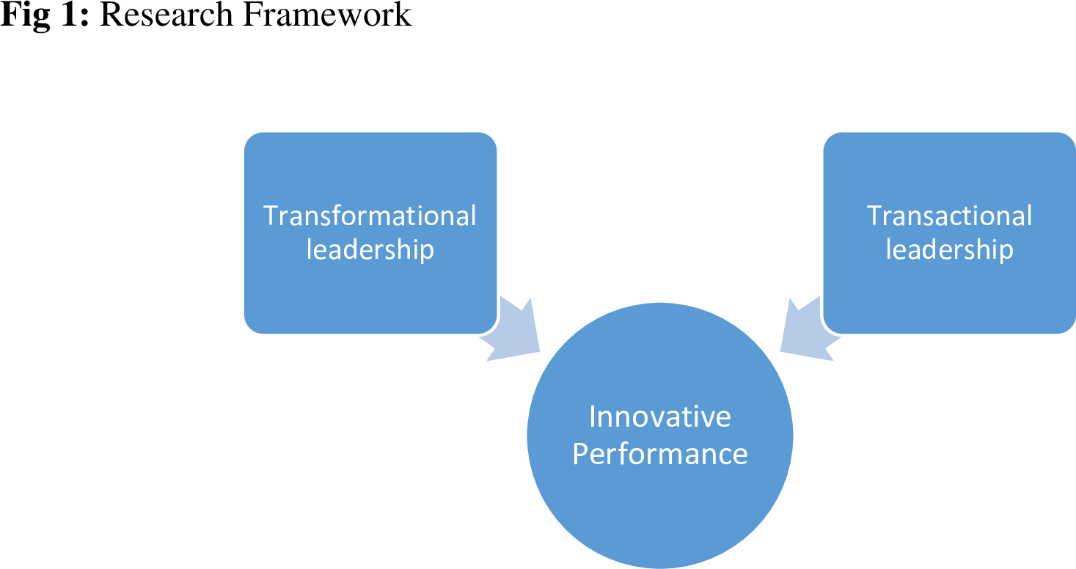
**

**
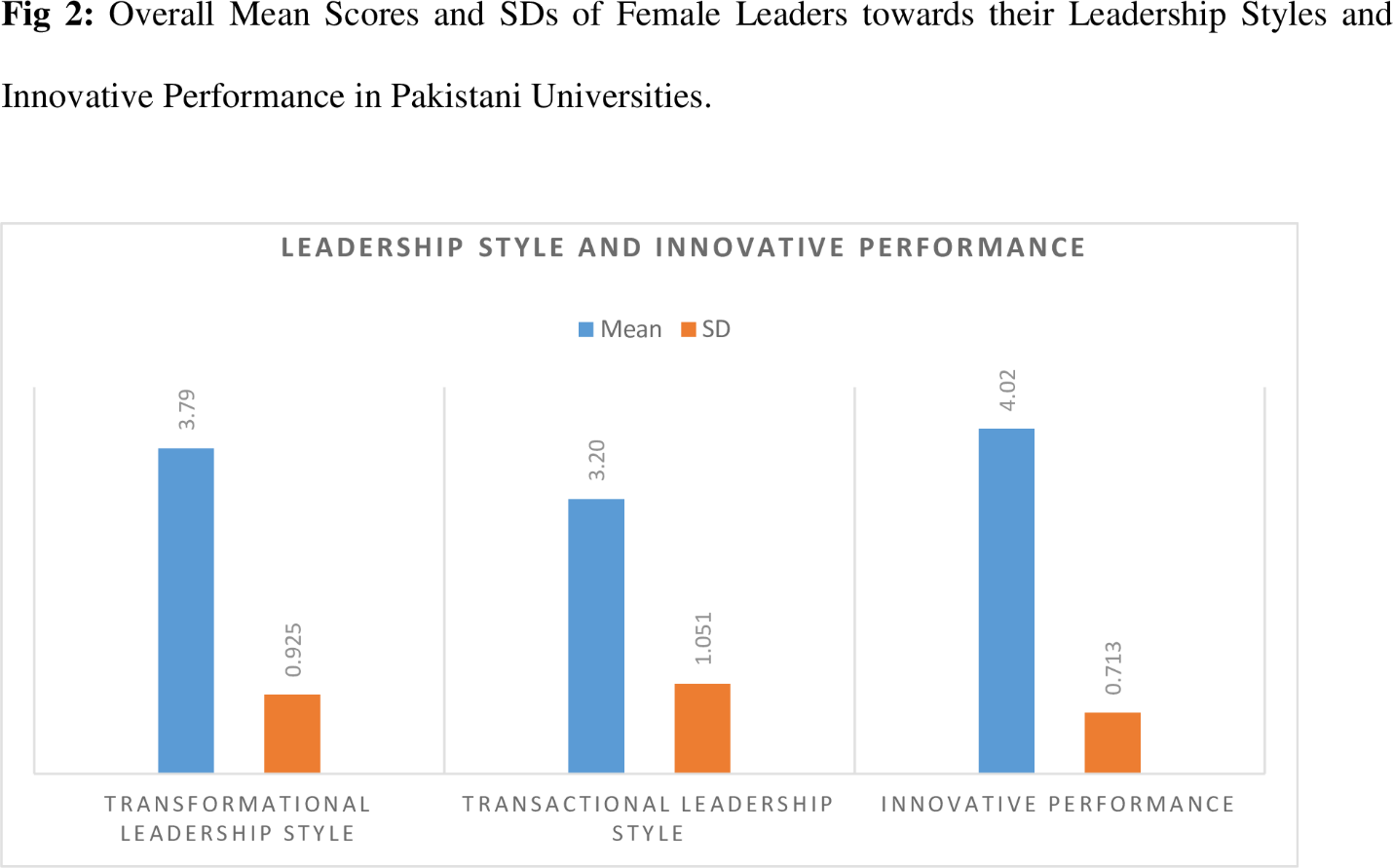
**

**
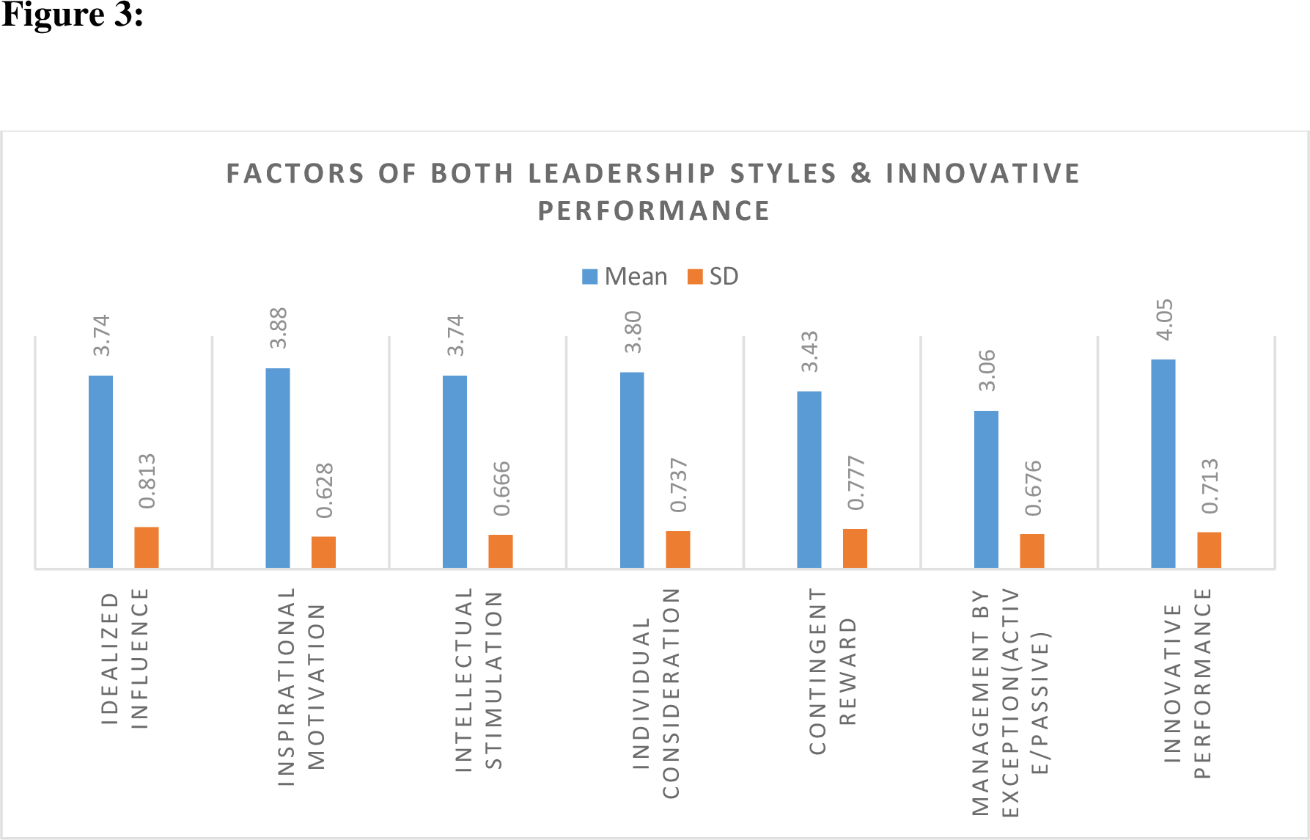
**

**Table 1:** Criteria to assess females' level of leadership style and innovative performance

|  | Mean Scores (*M*) | Perceiving degree |
| --- | --- | --- |
|  | Less than 1.8 | Very low |
|  | 1.8 to 2.5 | Low |
|  | 2.6 to 3.5 | Moderate |
|  | 3.6 to 4.2 | High |
|  | 4.3 and above | Very High |

**Table 2:** Mean Scores and SDs of Female Leaders towards their Leadership Styles and Innovative Performance in Pakistani Universities (N=100)

|  | **Variables** | **Mean** | **SD** | **Perceiving**  **degree** | **Rank** |
| --- | --- | --- | --- | --- | --- |
|  | Transformational Leadership style | 3.79 | 0.925 | High | 2 |
|  | Transactional Leadership style | 3.20 | 1.051 | Moderate | 3 |
|  | Innovative performance | 4.02 | 0.713 | High | 1 |

**Table 3:** Factor wise mean scores and SDs of transactional & transformational leadership styles of female leaders (N=100)

|  | **Factors** | **Mean** | **SD** | **Perceiving**  **degree** | **Rank** |
| --- | --- | --- | --- | --- | --- |
|  | **Transformational Leadership style** |  |  |  |  |
|  | Idealized Influence | 3.74 | 0.813 | High | 4 |
|  | Inspirational Motivation | 3.88 | 0.628 | High | 2 |
|  | Intellectual Stimulation | 3.74 | 0.666 | High | 5 |
|  | Individual Consideration | 3.80 | 0.737 | High | 3 |
|  | **Transactional Leadership style** |  |  |  |  |
|  | Contingent Reward | 3.43 | 0.777 | Moderate | 6 |
|  | Management by Exception (Active/Passive) | 3.06 | 0.676 | Moderate | 7 |
|  | **Innovative performance** | 4.05 | 0.713 | High | 1 |

**Table 4:** Relationship between Transactional Leadership and Innovative Performance

| **Variables** | | **Transactional** | **Innovative_ Performance** |
| --- | --- | --- | --- |
| Transactional | Pearson Correlation |  | .380^**^ |
|  | Sig. (2-tailed) |  | .000 |
|  | N | 100 | 100 |
| Innovative Performance | Pearson Correlation | .380^**^ |  |
|  | Sig. (2-tailed) | .000 |  |
|  | N | 100 | 100 |

**Table: 5** Relationship between Transformational Leadership and Innovative Performance

| **Variables** | | **Transformational** | **Innovative_ Performance** |
| --- | --- | --- | --- |
| **Transformational** | Pearson Correlation |  | .472^**^ |
|  | Sig. (2-tailed) |  | .000 |
|  | N | 100 | 100 |
| **Innovative Performance** | Pearson Correlation | .472^**^ |  |
|  | Sig. (2-tailed) | .000 |  |
|  | N | 100 | 100 |
